# 2781. The Use of Aminoglycosides for Treatment of Urinary Tract Infections in Pediatric Patients, a Carbapenem-Sparing Approach

**DOI:** 10.1093/ofid/ofad500.2392

**Published:** 2023-11-27

**Authors:** Jamie Legaspi, Kanokporn Mongkolrattanothai, Priya Edward, Regina Orbach

**Affiliations:** Children's Hospital Los Angeles, Los Angeles, California; Children Hospital Los Angeles, los angeles, CA; Children's Hospital Los Angeles, Los Angeles, California; Children's Hospital Los Angeles, Los Angeles, California

## Abstract

**Background:**

Urinary tract infections (UTIs) caused by antimicrobial-resistant pathogens have been increasingly reported. Aminoglycosides are known to be effective therapy for UTIs, however their use is not common due to a fear of nephrotoxicity. Overuse of broad-spectrum antimicrobial agents, particularly carbapenems can lead to colonization with carbapenem-resistant pathogens resulting in more difficult-to-treat infections. We aim to evaluate the use of aminoglycosides for UTI therapy in pediatric patients to assess the clinical outcome.

**Methods:**

A retrospective chart review was performed to evaluate all orders of aminoglycosides for the indication of UTI from 2020-2022 at Children’s Hospital Los Angeles. Demographic data, bacterial pathogen, kidney function before and during treatment, and clinical outcomes were collected and analyzed. We also evaluated the resistance patterns of bacterial pathogens, and whether they met our institutional criteria of a multi-drug resistant organism (MDRO). Patients were excluded if they received ≤ 48hrs of treatment with an aminoglycoside or if they did not have a gram-negative pathogen identified on urine culture.

**Results:**

A total of 61 patient encounters included an aminoglycoside order for treatment of UTI during the review period. 45 out of 61 encounters had a positive urinary culture that met our institutional criteria for an MDRO. Among patients receiving aminoglycosides, we observed 4 cases of elevated sCr that met our definition of AKI. The 2 cases with the greatest creatine increase (3-4 x baseline), were also currently receiving empiric vancomycin treatment. No patients had a documented treatment failure requiring change or escalation in therapy, or lack of resolution of fever. Four cases had a recurrence within 30 days after discharge. Three out of the four patients with recurrence had pre-existing anatomical abnormalities of the urinary tract that put them at increased risk for recurrent infection.

**Conclusion:**

Overall, extended interval aminoglycoside therapy was effective and well-tolerated for the treatment of urinary tract infections in our pediatric patient population. For cases of multi-drug resistant urinary pathogens, aminoglycosides represent a reasonable alternative to broader spectrum options such as carbapenems.

**Disclosures:**

**All Authors**: No reported disclosures

